# Supplementary material for: KIR+ CD8+ T Lymphocytes in Cancer Immunosurveillance and Patient Survival: Gene Expression Profiling
Source: Cancers (Basel). 2020 Oct 15;12(10):2991. doi: 10.3390/cancers12102991 (PMC7650600; doi:10.3390/cancers12102991)
Supplement: Supplementary file 1 [file cancers-12-02991-s001.zip › cancers-923400 supplementary final/Supplementary figs 1 and 2 2020-agosto-22.pdf]

Supplementary Figure S1

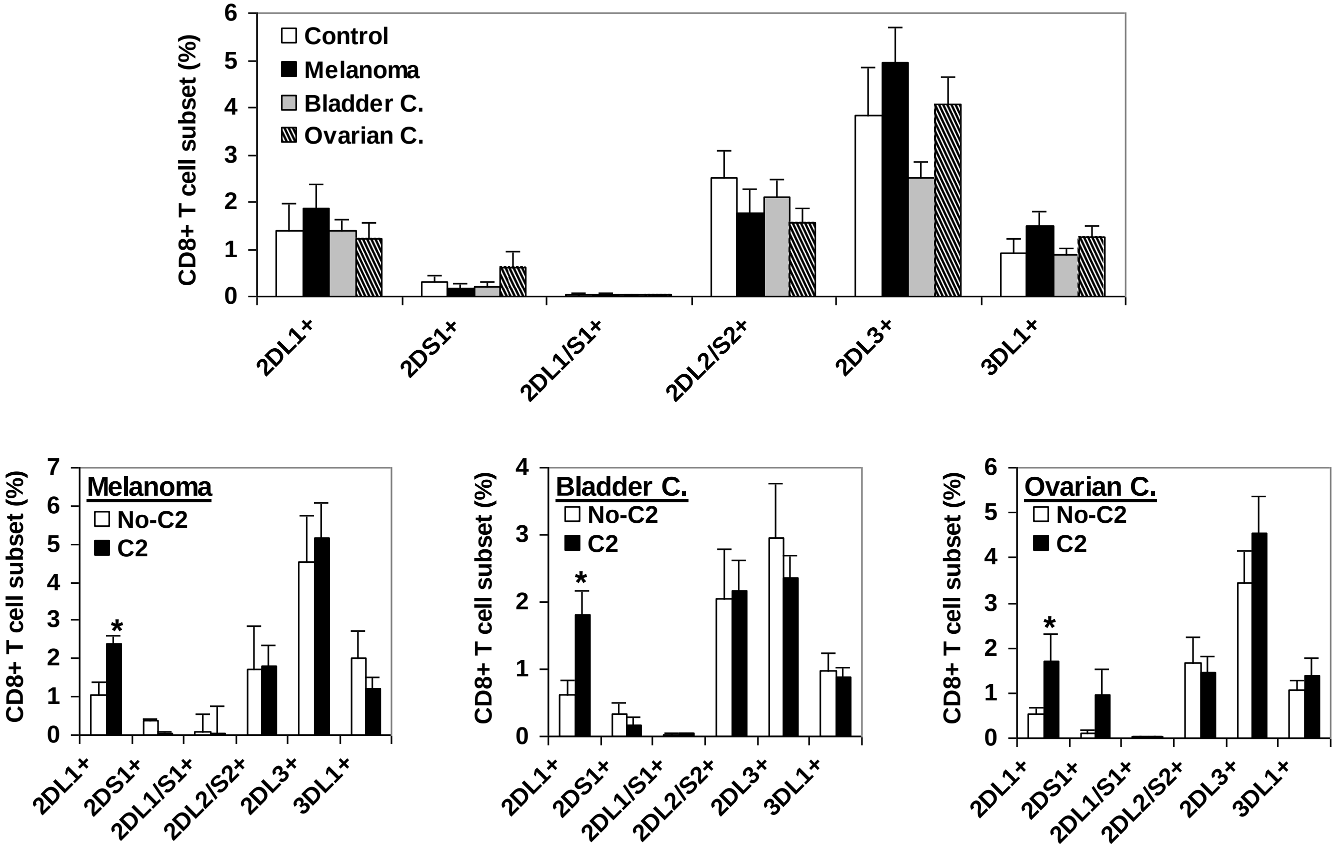

Supplementary Figure S1. Frequency of KIR+ CD8+ T cell subsets for Melanoma, Bladder, and Ovarian cancer patients. A, Frequency of KIR+ CD8+ T cell subsets in peripheral blood of healthy controls (n=42) and cancer patients (80 melanomas, 80 bladder cancers and 89 ovarian cancers) and according to the presence of the HLA-C2 or Bw4 ligands, respectively. * $p<0.05$, comparing KIR2DL1+ CD8+ T cells in HLA-C2 positive (C1C2 or C2C2) and negative (C1C1) cancer patients. No differences in KIR+ CD8+ T cell subsets were observed for HLA C1-ligands or Bw4-ligands in any of the three types of cancer (data not shown).

Supplementary Figure S2

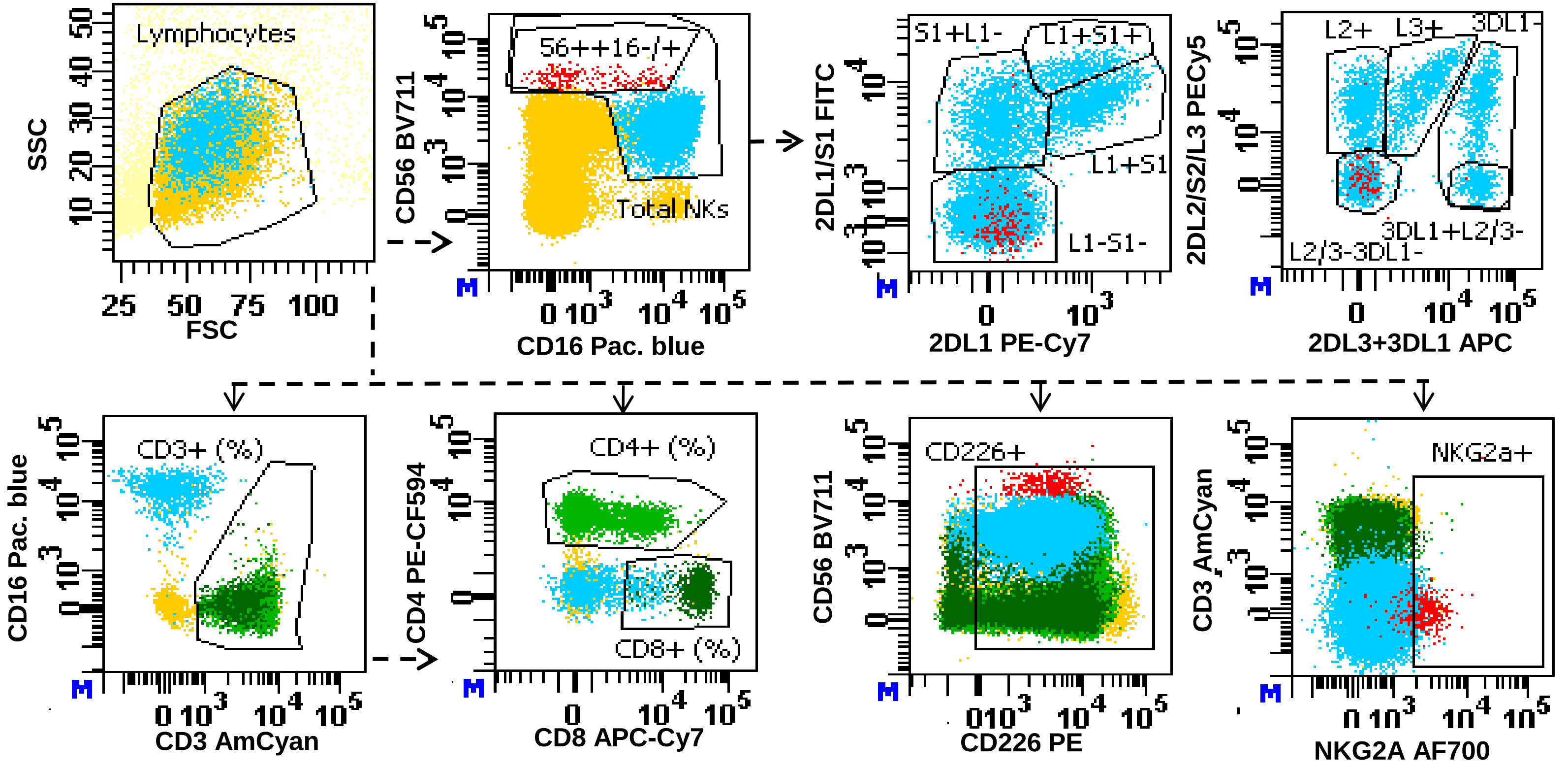

Supplementary Figure S2. Analysis of activating and inhibitory KIR receptors on peripheral blood CD8+ T cells and NK cells. Gating strategy to identify total lymphocytes, $CD3^+$, $CD4^+$ and $CD8^+$ T lymphocytes, $CD56^{++}CD16^{-/+}CD3^-$ ($CD56^{bright}$) and $CD56^+CD16^+CD3^-$ ($CD56^{dim}$) NK cells, $CD226^+$ cells, $NKG2A^+$ cells, as well as $KIR2DS1^+$, $KIR2DL1^+$, $KIR2DL1^+S1^+$, $KIR2DL2/S2^+$, $KIR2DL3^+$, and $KIR3DL1^+$ CD8+ T cells and NK cells. Diva software combines hierarchical and logical gating strategies to define different cell subsets (see tables below the figure). Lymphocytes were selected based on low dispersion for FSC/SSC and all lymphocyte subsets were hierarchically dependent on this gate. CD3+ lymphocytes were gated as $CD3^+CD16^{-/+}$ cells. $CD4^+$ and $CD8^+$ T cell subsets were set within $CD3^+$ lymphocyte. Total NKs gate included CD56+CD16+/- events combined with a NOT(CD3+) gate. To exclude M2-monocytes in the NK cell subset, $CD16^{++}CD56^-$ events were gated-out. The expression of KIR receptors was estimated on $CD3^+CD8^+$ T Lymphocytes ("CD8+") and on total NK cell ("NKs totales (%)") subsets. A special analysis described elsewhere [1] was applied to patients with KIR2DL3 alleles that were nonreactive with anti-KIR2DL3 antibody clone 180701 (KIR2DL3*005 or *015 alleles).

[1] Falco M, Romeo E, Marcenaro S, Martini S, Vitale M, Bottino C, et al. Combined genotypic and phenotypic killer cell Ig-like receptor analyses reveal KIR2DL3 alleles displaying unexpected monoclonal antibody reactivity: identification of the amino acid residues critical for staining. J Immunol 2010;185:433-41.

Tube: Tube_001 **Gating strategy**

Population	#Events	%Parent	%Total
All Events	505,859	####	100.0
Lymphocytes	388,043	76.7	76.7
CD3+	287,332	74.0	56.8
CD4+	231,088	80.4	45.7
CD8+	44,517	15.5	8.8
CD8+L1+	218	0.5	0.0
CD8+S1+	297	0.7	0.1
CD8+L1+S1+	20	0.0	0.0
CD8+3DL1+	241	0.5	0.0
CD8+L2+	1,588	3.6	0.3
CD8+L3+	3,298	7.4	0.7
Ts CD226+	272,443	94.8	53.9
CD4+CD226+ (%)	224,595	78.2	44.4
CD8+CD226+ (%)	37,480	13.0	7.4
Total NKs	60,766	15.7	12.0
NKG2a+	1,782	2.9	0.4
CD226+	59,680	98.2	11.8
NOT(CD3+ (%))	100,711	26.0	19.9
NKs totales (%)	59,746	15.4	11.8
56++16-/+	1,020	1.7	0.2
L2/3-3DL1-	23,727	39.7	4.7
L1-S1-	39,127	65.5	7.7
KIRneg	18,356	30.7	3.6
NKs CD226+	58,061	97.2	11.5
NKG2a++	5,732	9.6	1.1
NKG2a-	54,014	90.4	10.7
KIRnegNKG2a-	16,340	27.3	3.2
L1+S1-	7,064	11.8	1.4
S1+L1-	10,581	17.7	2.1
L1+S1+	2,907	4.9	0.6
3DL1+	13,642	22.8	2.7
L2/S2+	12,852	21.5	2.5
L3+	9,095	15.2	1.8
3DL1+L2/3-	5,314	8.9	1.1
L1+total	9,924	16.6	2.0
3DL1+L2/3-L1-S1-NKG2a-	3,425	5.7	0.7
L1+S1-L2/3-3DL1-NKG2a-	2,075	3.5	0.4
L2+L1-S1-3DL1-NKG2a-	7,236	12.1	1.4
L3+L1-S1-3DL1-NKG2a-	4,285	7.2	0.8
S1+L1-L2/3-3DL1-NKG2a-	2,401	4.0	0.5
16-56-	204,020	52.6	40.3
CD226-	26,416	6.8	5.2
Monocyte vs NKs CD56-CD16+	1,631	0.4	0.3
